# Smart Capture Modules for Direct Sensor-to-FPGA Interfaces

**DOI:** 10.3390/s151229878

**Published:** 2015-12-16

**Authors:** Óscar Oballe-Peinado, Fernando Vidal-Verdú, José A. Sánchez-Durán, Julián Castellanos-Ramos, José A. Hidalgo-López

**Affiliations:** 1 Departamento de Electrónica, Universidad de Málaga, Andalucía Tech, Campus de Teatinos, Málaga 29071, Spain; oballe@uma.es (O.O.-P.); jsd@uma.es (J.A.S.-D.); julian@elca.uma.es (J.C.-R.); jahidalgo@uma.es (J.A.H.-L.); 2 Instituto de Investigación Biomédica de Málaga (IBIMA), Málaga 29010, Spain

**Keywords:** direct sensor-to-digital device interface, FPGAs, parallel analog data acquisition

## Abstract

Direct sensor–digital device interfaces measure time dependent variables of simple circuits to implement analog-to-digital conversion. Field Programmable Gate Arrays (FPGAs) are devices whose hardware can be reconfigured to work in parallel. They usually do not have analog-to-digital converters, but have many general purpose I/O pins. Therefore, direct sensor-FPGA connection is a good choice in complex systems with many sensors because several capture modules can be implemented to perform parallel analog data acquisition. The possibility to work in parallel and with high frequency clock signals improves the bandwidth compared to sequential devices such as conventional microcontrollers. The price to pay is usually the resolution of measurements. This paper proposes capture modules implemented in an FPGA which are able to perform smart acquisition that filter noise and achieve high precision. A calibration technique is also proposed to improve accuracy. Resolutions of 12 effective number of bits are obtained for the reading of resistors in the range of an example piezoresistive tactile sensor.

## 1. Introduction

The incorporation of advanced technology into daily life requires complex smart systems able to face tasks in unstructured environments where events cannot be predicted. This means that smart systems that can acquire a huge amount of data from sensors and other processing devices have to be developed. Several solutions are possible to cope with this, and a tradeoff between throughput and complexity can determine which is chosen for a specific application.

The number of input channels is a key aspect in the complexity of the system. Analog sensors make up around 50% of the sensors on the market [1]. The common approach for data acquisition from analog sensors is some signal conditioning circuitry, mostly based on operational amplifiers, plus an analog to digital converter. As the number of sensors increases, *i.e.*, they become more complicated, several analog to digital converters are required. Microcontrollers usually have a multiplexer to share one analog to digital converter between a set of analog input channels [2]. Therefore, data acquisition is carried out in a sequential way. Since processing is also performed sequentially by the CPU of the microcontroller, the input-output delay to respond to a certain event increases. Therefore, the cost and size of the system also increases owing to the need for external signal conditioning circuitry. Finally, this realization is limited by the number of A/D channels of the microcontroller.

An alternative to the above approach is the direct sensor-microcontroller interface [3]. This approximation consists basically in measuring a time interval whose length is determined by the resistance or capacitance of a resistive or capacitive sensor respectively. Such implementation requires few external components. Regarding the microcontroller resources, a general purpose I/O pin can be used as input interface. However, to reduce error in measuring the time caused by other activity in the microcontroller, it must be kept in sleep mode or a specific capture module must be used. The former will obviously reduce the system throughput, while the latter is quite limited in number.

Such restrictions are overcome if a Field Programmable Gate Array (FPGA) is used instead of a microcontroller. The reason is that the internal hardware can be configured to have many blocks working in parallel. This has been exploited to execute complex processing algorithms in real time [4], and the same strategy can be applied to carry out massive parallel data acquisition from analog capacitive or resistive sensors [5].

The lack of Schmitt Trigger input buffers is a drawback of the FPGAs when compared to microcontrollers in the context of direct interface with sensors. The hysteresis of these buffers reduces uncertainty due to trigger noise associated with the detection of the instant when the input signal (with a slow slew rate) crosses the threshold of the input buffer. This event signals the end of the time interval to be measured, so trigger noise limits the precision of measurement. Moreover, the threshold $V_{IL}$ related to low logical value of the Schmitt Trigger input buffers is more robust against noise superimposed on the power supply [6].

The proposal in [7] introduces hysteresis by implementing a block composed of an input buffer, an output buffer to add positive feedback, and two external resistors. Together with the RC network R or C can both be the sensor) the resulting circuit is a relaxation oscillator whose output waveform, *i.e.*, its period or the duration of a semi-cycle, can be used to obtain the measurand value. The hysteresis size can be controlled by the resistance of the external resistors. Though robust to trigger noise, the input impedance of the implemented block is low, and the voltage excursion at the output node of the RC network is limited by the hysteresis value. As a consequence, the dynamic range is not clearly improved by this strategy.

This paper presents some capture input blocks implemented in an FPGA. They take the input from the common RC networks used in direct interfaces and provide an output to signal the end of the time interval to be measured. The resources in the FPGA allow digital circuits to be built that detect the first change of logical value at the input buffer when the input signal reaches the threshold. Instead of adding analog positive feedback [7], such feedback is implemented in digital circuits to achieve the memory of the hysteresis cycle. Moreover, smarter capture modules can be used to improve performance and precision. For instance, modules that are robust enough to isolated glitches in the input signal. In addition, the flexibility of the storage elements in the FPGA to be synchronized with both edges of the clock signal, and also the detection of, not only the first but the last transition at the output of the input buffer is exploited to carry out averaging. This actually filters part of the trigger noise and achieves more precision without losing bandwidth.

Regarding accuracy, it is mainly limited by the impedance of the input buffers of the FPGA [8,9]. Single-point or two-point calibration techniques can be used to improve accuracy. They require one external resistor and one additional FPGA pin, or two external resistors plus two additional FPGA pins, respectively. This paper compares the performance obtained with both methods. Moreover, it explores the implementation of another strategy that stores parameters obtained from previous characterization of the input buffers in the FPGA. Such strategy uses less external resources than the two-point technique and achieves similar accuracy, though it requires a previous characterization step.

## 2. Direct Interface with Sensors

**Figure 1:**
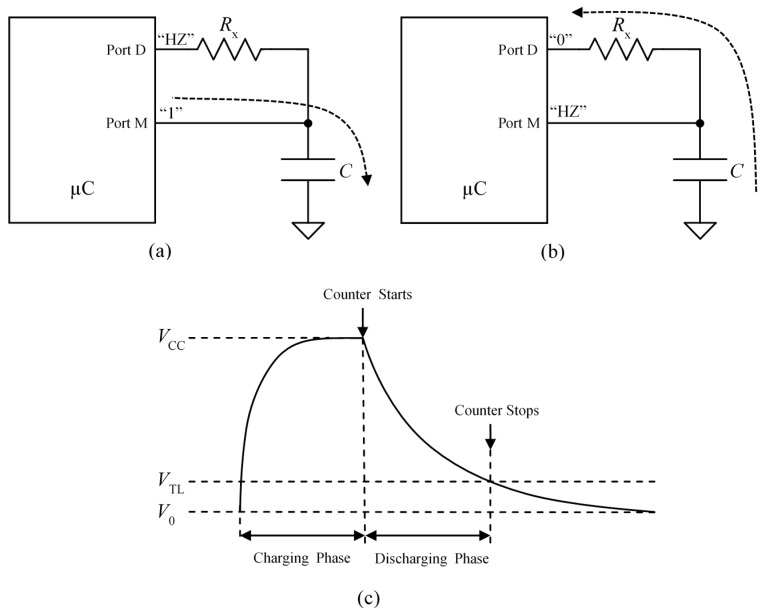
(**a**) Direct interface configured to charge the capacitor; (**b**) direct interface configured to discharge the capacitor and monitor its voltage drop; and (**c**) voltage drop in the capacitor *versus* time.

Figure 1 illustrates the procedure to implement direct interfaces between digital input ports and capacitive or resistive sensors. The measurement is taken in two steps. Firstly, the capacitor C is charged through the port M configured as output buffer with output corresponding to a logical value “1” (see Figure 1a). Secondly, the capacitor is discharged through the resistor $R_{x}$ and the port D configured as output buffer with output corresponding to a logical value “0” (see Figure 1b). The measurand value is related to the time it takes to discharge the capacitor, and it can be obtained if the voltage drop in it is monitored. This can be done with the Port M configured as high impedance (HZ) input buffer. The internal output node of this buffer changes to high logical value when its input reaches the low threshold voltage $V_{TL}$ of the input buffer. Assuming that the capacitor has been charged up to the voltage supply $V_{CC}$ in the first step (Figure 1a), the time interval between the beginning of the second step in Figure 1b and this event is

(1) $$T_{x}=R_{x}C\ln\left(\frac{V_{CC}-V_{0}}{V_{TL}-V_{0}}\right)$$

Therefore, provided we know $V_{CC}$, $V_{TL}$, $V_{0}$ (usually 0 volts) and C we can obtain the value of the resistance $R_{x}$ from $T_{x}$ in Equation (1). $T_{x}$ is readily measured with a high frequency clock and a timer whose count is registered when the threshold $V_{TL}$ is reached.

### 2.1. Precision

The statistical spread of the results of this count provided by different measurements for the same resistance is the uncertainty or precision. The sources of uncertainty are the quantization error, the instability of the reference oscillator to generate the clock signal, and the trigger noise or noise added to the input signal and the threshold $V_{TL}$. If only the quantization error is taken into account, the precision expressed in number of bits M is

(2) $$M=\text{lb}\left(\frac{T_{x,\max}-T_{x,\min}}{T_{s}}\right)$$

where $T_{x,\max}$ and $T_{x,\min}$ are the maximum and minimum values of $T_{x}$ for the maximum and minimum values of the measurand $R_{x}$ respectively, and $T_{s}$ is the period of the clock signal. The precision *M* in Equation (2) is the maximum obtained under ideal conditions. If the other sources of uncertainty are taken into account, the precision is lower and it is expressed in the effective number of bits ENOB. If we use the terminology given in [10], the discharging time $T_{x}$ is the measurand Y and the resulting digital number multiplied by *T_s_* is the observed input quantity *X*. The relationship between Y and X is modeled by Y=X+Z, where Z takes into account the quantization effects. As the beginning of the interval is synchronized with the start of the counting, the quantization error in time is $0\leq EQ\leq T_{s}$. Then Z is described by a rectangular probability density function, and its standard uncertainty is $u(z)=T_{s}/\sqrt{12}$. Thus, the ENOB can be obtained as [11]

(3) $$\text{ENOB}=M-\text{lb}\left(\frac{u_{\max}(y)}{u(z)}\right)$$

where

(4) $$u(y)=\sqrt{u^{2}(x)+u^{2}(z)}$$

The term *u*(x) models the noise in the measurement with the circuit in Figure 1 [11].

### 2.2. Accuracy

The values of $V_{CC}$, $V_{TL}$ or C in Equation (1) change with time and temperature. Therefore, a calibration procedure should be implemented to compensate for these sources of error in the measurement of $T_{x}$. A straightforward strategy consists in measuring the discharging time $T_{c1}$ corresponding to a known resistance value $R_{c1}$, then the value of the unknown resistance $R_{x}$ is obtained as

(5) $$R_{x}^{*}=\frac{T_{x}}{T_{c1}}R_{c1}=\frac{N_{x}T_{s}}{N_{c1}T_{s}}R_{c1}=\frac{N_{x}}{N_{c1}}R_{c1}$$

where $N_{x}$ and $N_{c1}$ are the integer numbers produced during the discharging through $R_{x}$ and $R_{c1}$, respectively.

If the measurements of $T_{c1}$ and $T_{x}$ are taken under similar conditions, the multiplicative interference is the same for both and is cancelled in Equation (5). This technique is called single point calibration.

**Figure 2:**
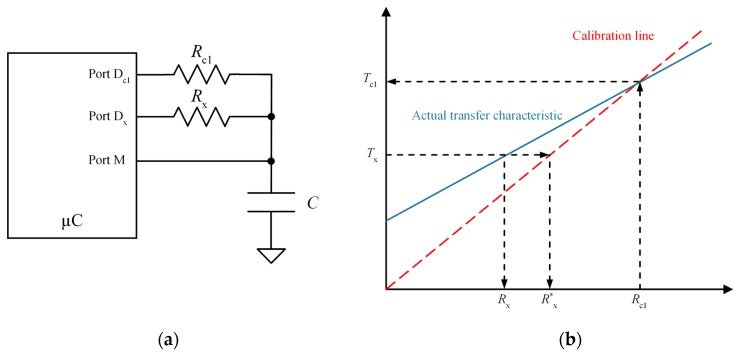
Single point calibration: (**a**) circuit and (**b**) actual and calibration transfer characteristics.

However, Equation (5) does not take into account the input resistance of the ports D_*c*1_ and D_*x*_ in Figure 2a. If these resistances are considered as constants, the actual transfer characteristic is not that given by Equation (5), depicted by a dashed, red line in Figure 2b, but by a solid blue one. Therefore, there is a difference between the value $R_{x}^{*}$ obtained from Equation (5) and the actual value $R_{x}$.

**Figure 3:**
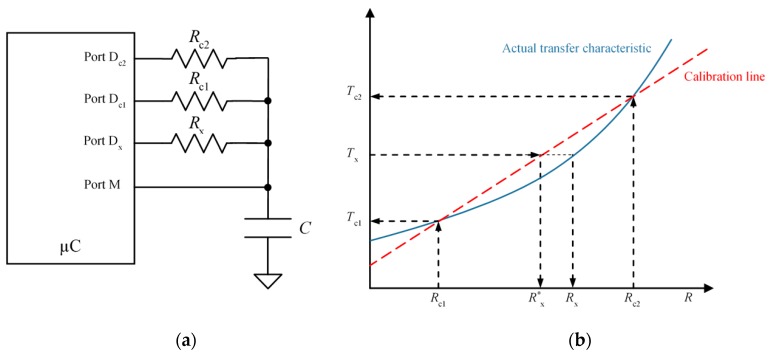
Two-point calibration technique: (**a**) circuit and (**b**) actual and calibration transfer characteristics.

Figure 3 illustrates the two-point calibration procedure based on the measurement of two known resistance values $R_{c1}$ and $R_{c2}$. The unknown resistance is then calculated as:

(6) $$R_{x}^{*}=\frac{N_{x}-N_{c1}}{N_{c2}-N_{c1}}\left(R_{c2}-R_{c1}\right)+R_{c1}$$

where $N_{c1}$ and $N_{c2}$ are the digital numbers of the discharging times for the calibration resistances $R_{c1}$ and $R_{c2}$, respectively. The red dashed line in Figure 3b represents the calibration line in Equation (6), and the solid line represents the actual transfer characteristic if the input resistance of the ports in Figure 3a is taken into account. The error is now minimum for $R_{x}=R_{c1}$ and $R_{x}=R_{c2}$. This error is now of the order of the difference of the input resistances of D_*c*1_, D_*c*2_ and D_*x*_ in Figure 3a, while it is of the order of the absolute value of the input resistance of ports D_*c*1_ and D_*x*_ in Figure 2a, therefore the zero and sensitivity errors are smaller. This paper proposes a method that achieves similar accuracy to that obtained with the two-point calibration technique but with only one calibration resistor.

## 3. Capture Modules on FPGAs

**Figure 4:**
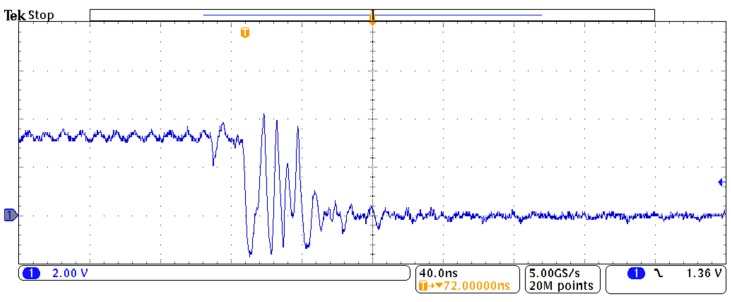
Transitory at the output of the input buffer when the threshold $V_{IL}$ is reached (routed through another buffer to a port of the FPGA to be measured).

If a crystal oscillator, with a stability greater than 50–100 × 10^−6^ [11], is used to generate the clock signal, the precision is mainly limited by the quantization and the trigger noise. The latter has three components: the noise superimposed on the input signal; the noise superimposed on the threshold $V_{TL}$; and the noise on the capacitor at the end of the charging stage. These sources of noise make the internal output of the input buffer of port M in Figure 2 oscillate when the input signal reaches the threshold (see Figure 4). Therefore, there is not only one event that signals the end of the count to measure $T_{x}$, but many. In the following, different capture modules are proposed to detect the end of the count taking into account the noisy transition in Figure 4.

### 3.1. Capture Module 1 (CM1)—Synchronous Circuit to Signal the Input Fall Edge with a Pulse

**Figure 5:**
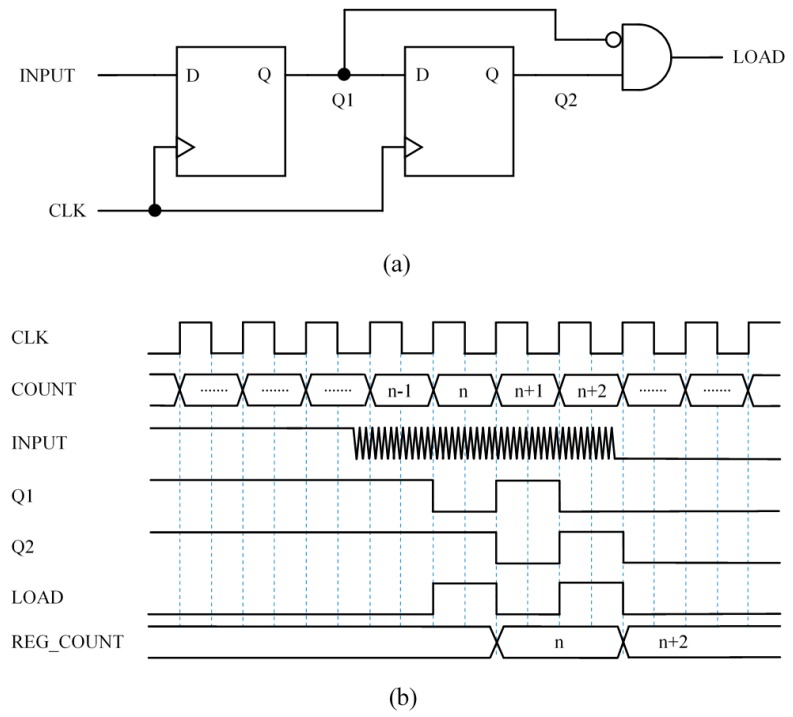
Capture Module 1—Synchronous circuit to signal the input fall edge with a pulse: (**a**) circuit and (**b**) example chronogram.

Figure 5 shows a simple way to detect a falling edge at the input signal and generate a pulse of one period of the clock signal duration (signal LOAD at Figure 5b). When this pulse is detected, the content of the timer is stored in the register REG_COUNT. However, since the input signal is noisy, the circuit does not generate only one pulse but many (see Figure 5b), and the count stored in REG_COUNT is the one corresponding to the last pulse. Note that the pulse at the LOAD signal is not generated at the first rising edge of the clock after the input changes. This is because the two signals are not synchronized and the input signal has to have a low logical value at the rising edge of the clock signal. Since trigger noise makes this input oscillate, it is uncertain at what clock cycle the event will be captured.

### 3.2. Capture Module 2 (CM2)—Synchronous Circuit to Signal the Input Fall Edge with a Single Pulse

**Figure 6:**
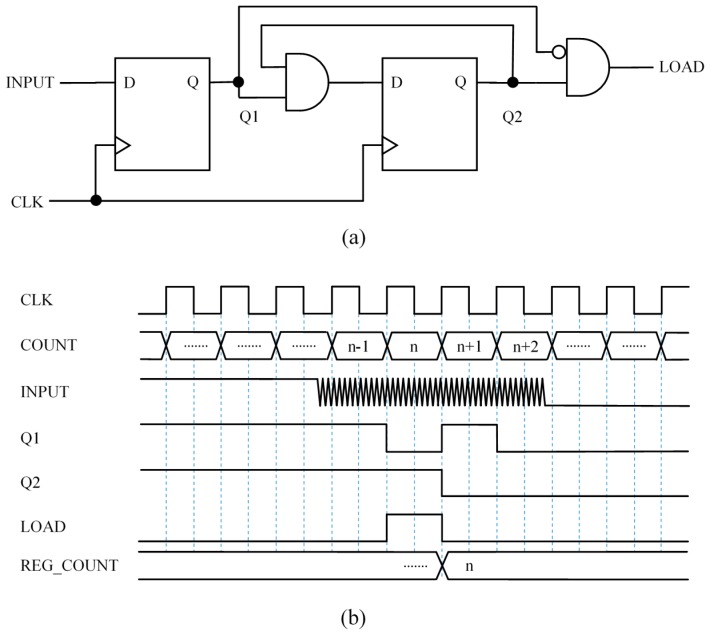
Capture Module 2—Synchronous circuit to signal the input fall edge with a single pulse: (**a**) circuit and (**b**) example chronogram.

The circuit in Figure 6a slightly modifies that in Figure 5a by adding feedback. Note that once Q2 has changed to logical low value the input of the second flip-flop is set to low value so Q2 will be a logical “0” until the inputs of the flip-flops are set to high value by a preset signal, and the circuit is ready to detect another falling edge at the input.

The digital feedback introduces memory and the circuit is able to store a bit that indicates that a falling edge at the input has already been detected. This memory actually replaces the analog memory present in proposals with analog circuits with hysteresis, such as Trigger Schmitt buffers or the proposal in [7].

Figure 6b shows a chronogram to illustrate how this circuits works. Again, the uncertainty due to the lack of synchronization between the input and clock signals is present, and the pulse at the LOAD signal is generated at the second rising edge of the clock after the input signal begins to oscillate.

### 3.3. Capture Module 3 (CM3)—Circuit Based on Front End Interface with Latch

**Figure 7:**
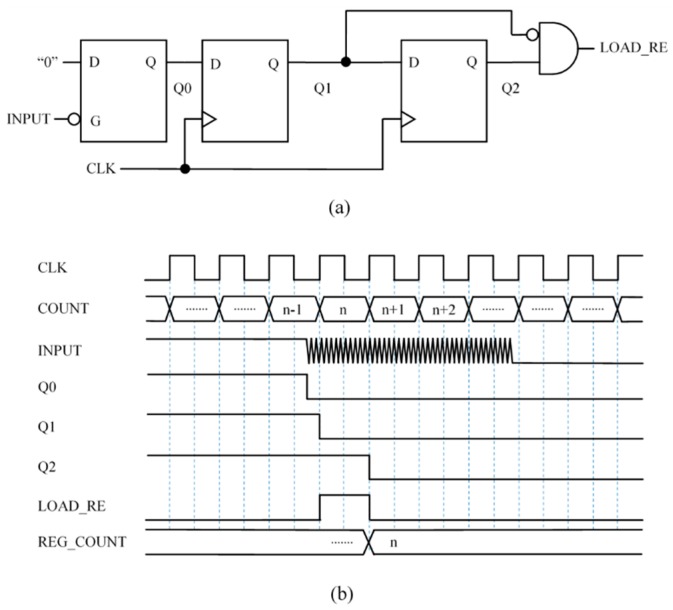
Capture Module 3—Circuit based on front end interface with latch: (**a**) circuit and (**b**) example chronogram.

Figure 7a shows another way to add memory like that present in the hysteresis of Schmitt Trigger buffers to the basic circuit in Figure 5a. The circuit exploits the flexibility of the FPGA to design with different storage elements and uses a level triggered latch to store one bit. Specifically, when the input signal changes to logical low value because the voltage drop in the capacitor reaches $V_{TL}$, the latch stores the logical “0”. Note that the Q0 keeps the value whatever the following value of the input is, so the circuit provides a single input at LOAD_RE after the first transition from high to low value of the input.

### 3.4. Capture Module 4 (CM4)—Circuit Based on Front End Interface with Latch Robust against Spikes

**Figure 8:**
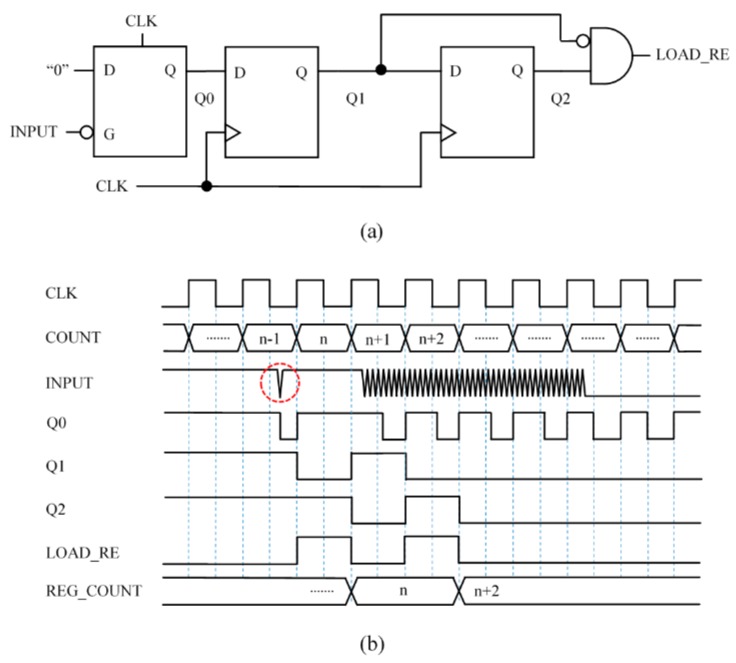
Capture Module 4—Circuit based on front end interface with latch robust against spikes: (**a**) circuit and (**b**) chronogram.

A drawback of the circuit in Figure 7a is that it is sensitive to spurious transitions to low value or glitches at the input due to isolated noise spikes. The circuit in Figure 8a is more robust against such events. It uses the clock signal to preset the input latch every clock cycle, at the high semi-cycle. Therefore, if there is a short glitch at INPUT, Q0 will not keep the low value and the circuit will be ready to generate another pulse at the output to register the content of the timer. However, since Q1 does not change until the rising edge of the clock signal, a low value of the input in the preceding low semi-cycle will set a “0” again at Q0 and Q1 will not change, so there will be no pulse at the circuit output. In other words, if INPUT is at high value at least for a high clock semi-cycle then the glitch is considered as noise caused by an isolated spike and a new LOAD_RE event could be generated. Therefore, the capture module in Figure 8a provides a load pulse for the first isolate transition to low at the input caused by the trigger noise, but discards it with a new pulse when the oscillation starts. As a result, the circuit reduces the uncertainty in determining $T_{x}$.

### 3.5. Capture Module 5 (CM5)—Circuit Triggered with the Rising and Falling Edge of the Clock Signal to Average Two Counts

**Figure 9:**
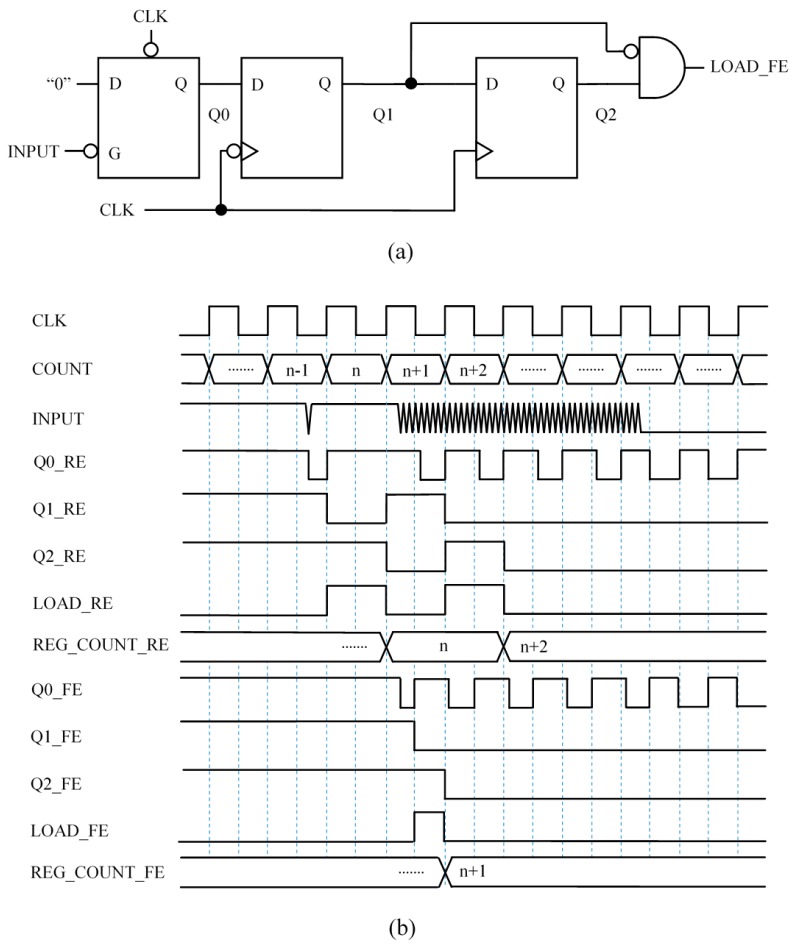
Capture Module 5—circuit triggered with the rising and falling edge of the clock signal to average two counts: (**a**) circuit and (**b**) example chronogram.

Another interesting feature of the FPGAs is the possibility to use flip-flops triggered by rising or falling edges. Figure 9a shows a circuit similar to that in Figure 8a but with the flip-flop whose outputs Q1 are triggered by a falling edge, and the latch preset at the low semi-cycle of the clock signal. Figure 9b shows a possible chronogram associated to both circuits. This is only an example, the output pulses of both circuits can be separated in time. The reason is that the input signal is complex and is not synchronized with the clock. However, we observe experimentally that if we use both circuits in parallel and the readings of the timer are stored in two registers, their average provides a more precise value of $T_{x}$. An explanation for this is the ability to store the count in the timer with a higher resolution in time, actually with twice the frequency of the clock. Therefore, although the time base does not change, the effect is similar to reduce the quantization error. This is the case in Figure 9b, where two consecutive counts (n + 1 and n + 2) are stored, so their average will provide a higher resolution. Another explanation for this improvement in the precision is the low pass filtering implemented by the average. The output pulses in LOAD, or the counts registered, could be not consecutive.

### 3.6. Capture Module 6 (CM6)—Circuit Triggered with the Rising and Falling Edge of the Clock Signal to Average Four Counts

The above mentioned filtering function performed by the average can be exploited to improve the precision of measurement while preserving the bandwidth thanks to the parallel operation in the FPGA. Note that the voltage trigger noise is translated into a trigger noise in time, therefore an average in time of this noise will filter part of the noise. This can be done if circuits which detect the end of the noisy change at the input of the capture modules are proposed and developed. Then the counts registered by the circuits that signal the start of the noisy transition at the input and those registered by the circuits that detect the end of this noisy transition are averaged.

**Figure 10:**
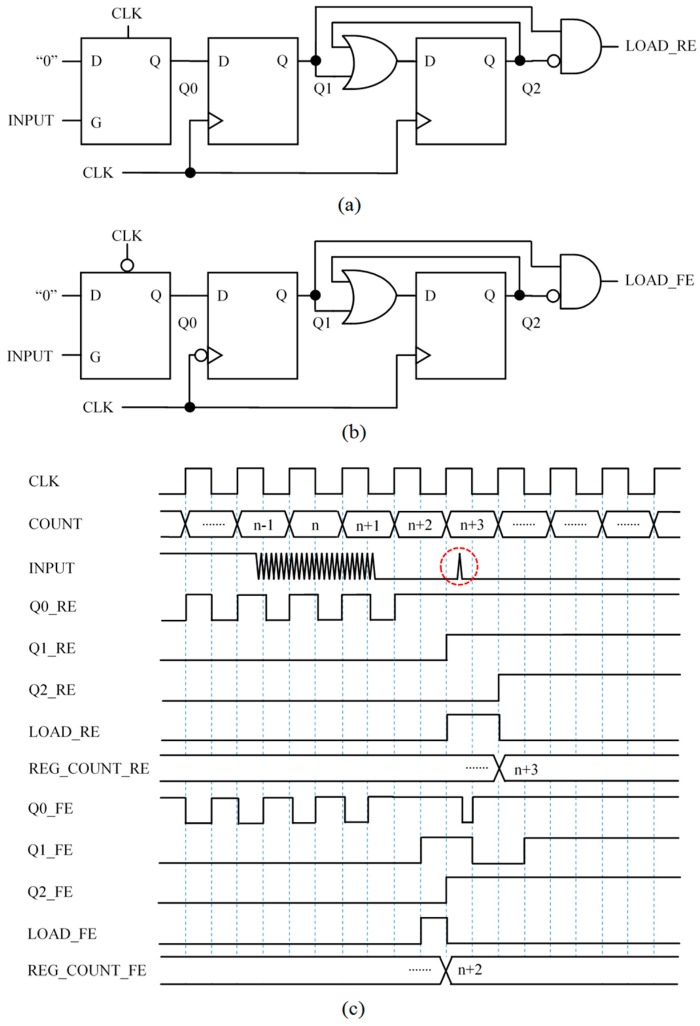
Capture Module 6—circuit triggered with the rising and falling edge of the clock signal to average four counts: (**a**) circuit triggered with the rising edge; (**b**) circuit triggered with the falling edge; and (**c**) chronogram.

Figure 10a shows a circuit able to detect the end of the noisy transition at the input with flip-flops synchronized with the rising edge of the clock signal. The circuit works as follows. Since the clock signal makes a PRESET every clock cycle, the signal Q0 toggles every clock cycle as long as the INPUT signal takes a high logical value at least once in the low semi-cycle. However, as soon as the INPUT signal stays stable at low logical value, Q0 also stabilizes at high logical value. Then a pulse is generated at the circuit output LOAD_RE. To make the circuit robust against isolated noise spikes, a feedback with a logical gate OR is added at the last flip-flop whose output is Q2. This causes the logical high value to be stored at Q2 whatever the value of Q1 is, as long as there is not a logical one at INPUT in the low semi-cycle of the clock signal prior to the clock rising edge. Note that, in Figure 10c, the last isolated glitch (red dotted circle) is considered as noise and it is not taken into account by this capture module.

Similarly to the proposal in Section 3.5, another circuit synchronized with the clock falling edge can be used in parallel with that in Figure 10a to improve the precision in the detection of the end of the noisy transition. This circuit is shown in Figure 10b. In this way the average of four registered counts (from the circuits in Figure 9 and Figure 10) is calculated and the result is more precise than the measurement of $T_{x}$ obtained with the other capture modules, as can be seen in Section 6.

## 4. Proposed Calibration Techniques

In addition to the capture modules to improve resolution described above, this paper also proposes calibration techniques with higher accuracy. As mentioned in Section 2.1, error estimation of resistance $R_{x}$ is mainly due to the input resistance of the ports of the FPGA that drive $R_{x}$, as well as the calibration R_*c*1_. If the input internal resistance of the ports is taken into account, the result of Equation (5) is not $R_{x}$ but [12]

(7) $$R_{x}^{*}=\frac{N_{x}}{N_{c1}}R_{c1}=\frac{R_{c1}}{R_{c1}+R_{n,c1}}R_{x}+\frac{R_{c1}R_{n,x}}{R_{c1}+R_{n,c1}}$$

where *R_n,x_* and *R_n,c1_* are the internal resistances of the ports D_*c*1_ and D*_x_* in Figure 2, respectively, the following relationship is readily obtained from Equations (5) and (7) between the actual value of the measurand $R_{x}$ and the ratio $N_{x}/N_{c1}$

(8) $$R_{x}=\left(\frac{N_{x}}{N_{c1}}R_{c1}-\frac{R_{c1}R_{n,x}}{R_{c1}+R_{n,c1}}\right)\frac{R_{c1}R_{n,c1}}{R_{c1}}=\frac{N_{x}}{N_{c1}}\left(R_{c1}+R_{n,c1}\right)-R_{n,x}$$

Therefore, the measurement of the input resistances ($R_{n,c1}$ and $R_{n,x}$) obtains a more accurate value of $R_{x}$ from Equation (8). This measurement can be done with an ad-hoc circuit, or the values of $R_{n,c1}$ and $R_{n,x}$ can be estimated with the same direct interface that is depicted in Figure 2a. Note that Equation (8) corresponds to a line whose slope and zero are determined by the value of the internal and calibration resistances. Therefore, if the slope and zero are found, Equation (8) provides the “actual” value of $R_{x}$. These are readily obtained if we measure two known resistances, because in this way two equations are obtained where the slope and zero are the unknown parameters. This procedure will be referred to as “calibration with two-point linear characterization” (CLchar2).

**Figure 11:**
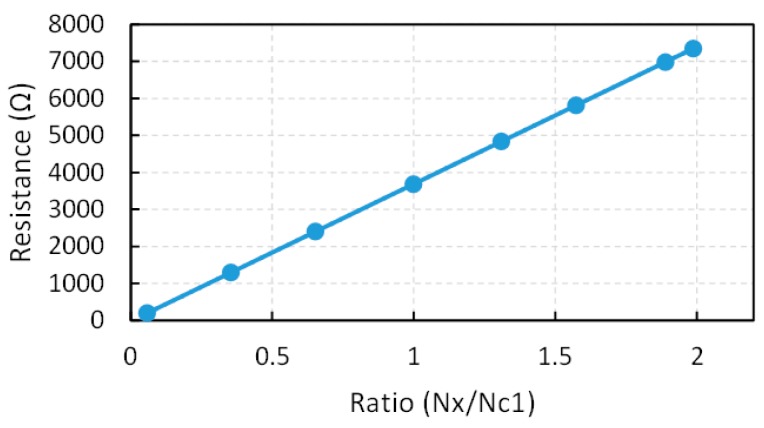
Experimental transfer characteristic with linear interpolation used to obtain Equation (9).

This technique can be generalized to contemplate a more precise linear approximation or a possible non-linear dependence of $R_{x}$ on the ratio $N_{x}/N_{c1}$ due to a more accurate model of the input impedance of the ports. This has also been done in this paper, where eight resistances of known value have been measured with the circuit in Figure 2a. Figure 11 depicts the resistance $R_{x}$ *versus* the ratio $N_{x}/N_{c1}$ for the measured resistances and also the linear approximation obtained by the least mean square regression as

(9) $$R_{x}=3704.79\frac{N_{x}}{N_{c1}}-14.07$$

which provides the “actual” value of *R_x_* for a given ratio $N_{x}/N_{c1}$. This is referred to as “technique calibration with 8-point linear characterization” (CLchar8). Finally, if a two degree polynomial fit is carried out from the same eight points, the expression for *R_x_* is

(10) $$R_{x}=-0.31{\left(\frac{N_{x}}{N_{c1}}\right)}^{2}+3705.45\frac{N_{x}}{N_{c1}}-14.28$$

This technique is referred to as “calibration with 8-point square characterization” (CSQchar8). All these approximations are used to improve accuracy in the measurement of $R_{x}$ and the results are shown in Section 6.

## 5. Materials and Methods

The circuit to test the proposals of this paper is based on an FPGA Spartan3AN from Xilinx (XC3S50AN-4TQG144C) [13]. The clock signal is generated with a crystal oscillator at 50 MHz. The circuit is implemented in a four layer FR4 printed circuit board. The design rules recommended by the vendor of the FPGA for the PCB are carefully followed to minimize the noise superimposed on the supply voltage because it couples with the threshold voltage $V_{TL}$ and to $V_{CC}$ in Equation (1) and hence contributes to the trigger noise and degrades precision. The FPGA works with two different supply voltages, one for the core and the other for the I/O buffers. Note that this fact reduces the noise contribution of the activity in the core of the FPGA on the voltage supply of the output buffers, so it reduces the noise added to $V_{CC}$ and $V_{TL}$ in Equation (1). The voltage regulators chosen for both supplies are the TPS79633 and the TPS79912 from Texas Instruments that provide 3.3 V and 1.2 V, respectively. Both have low dropout voltages and very low output voltage noise (40 µV_RMS_). A set of decoupling capacitors of different values are connected between voltage supply and ground pins (four supply pins for the I/O buffers and one for the core). The capacitors are physically close to the supply pins. The inner layers of the PCB are dedicated to the ground plane and the 3.3 V voltage supply plane. Note that the supply plane is connected to the voltage supply of the I/O buffers, since the noise superimposed on this voltage degrades the precision.

Two target devices are used to show the performance of the proposed modules: a custom piezoresistive tactile sensor; and a PT-1000 temperature sensor. Piezoresistive tactile sensors are basically arrays of force sensing resistors which are considered suitable to illustrate the proposals of this paper. The PT-1000 sensor also allows different output ranges to be tested as well as allowing a comparison of performance with other reported implementations.

For the results in Section 6 related to precision, the timers to measure $T_{x}$ have 14 bits and their time base is 20 ns. Since trigger noise mainly affects the measurement of low slew rate signals, the largest resistance in the range of interest is chosen to assess the performance of the proposal. The resistance is measured 500 times with the circuit in Figure 1 and the standard deviation σ(x) is calculated to estimate u(x). A capacitor of 47 nF was used since a larger capacitance does not improve precision significantly [11] and reduces bandwidth and increases power consumption.

**Figure 12:**
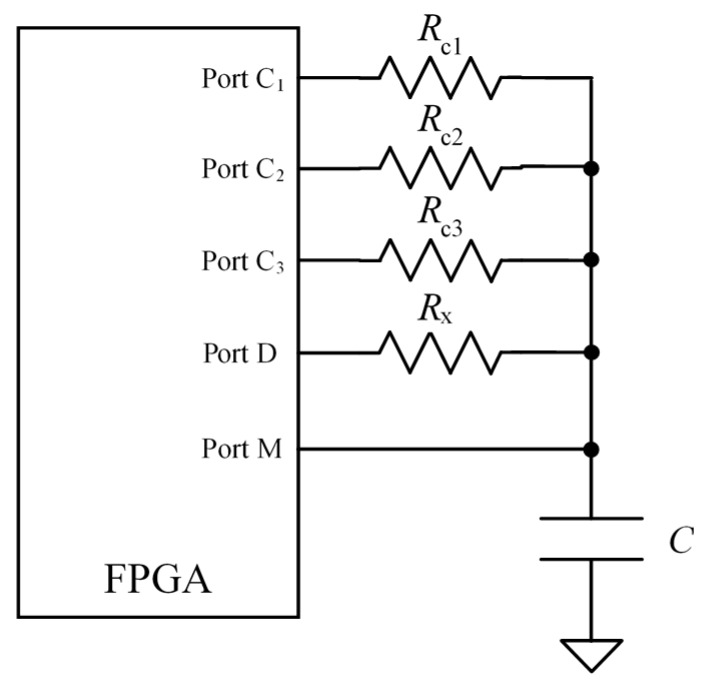
Circuit to implement and compare different calibration techniques.

Regarding accuracy, the circuit in Figure 12 is used to implement and compare the results of three calibration techniques: one-point calibration; two-point calibration; and the proposed technique described in Section 4. The values of the resistances $R_{c1}$, $R_{c2}$ and $R_{c3}$ in Figure 12 are taken with values in 50%, 15%, and 85% of the range of interest respectively [14]. Another set of known resistances is used to characterize input impedance of the ports of the FPGA as well as to assess the proposed technique and compare it with the others. The “actual” value of these resistances is measured with a digital multimeter (Agilent 34401) with an accuracy of 0.011% in the range of interest. Finally, the capture module implemented is that described in the Section 3.6.

## 6. Results and Discussion

**Figure 13:**
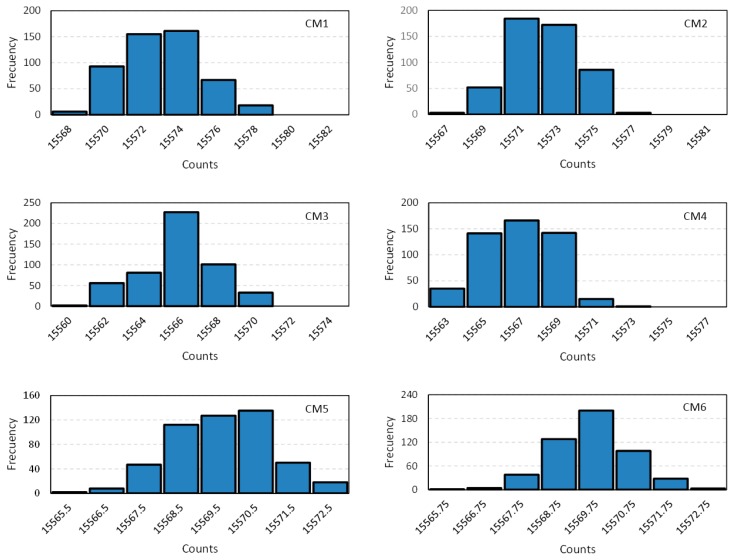
Histograms of 500 digital numbers for $R_{x}$ = 7350 Ω and C = 47 nF and for the capture modules: CM1 in Section 3.1, CM2 in Section 3.2, CM3 in Section 3.3, CM4 in Section 3.4, CM5 in Section 3.5 and CM6 in Section 3.6.

To test and compare the performance of the capture modules proposed in Section 3, the range of resistance of the piezoresistive tactile sensor between 200 Ω and 7350 Ω was chosen. Figure 13 shows the histograms of 500 digital numbers for $R_{x}$ = 7350 Ω and C = 47 nF, and for the capture modules described in Section 3. Moreover, Table 1 summarizes the data related to the precision obtained with the capture modules. It shows the results for the standard deviation σ(x), the uncertainty u(y) given by Equation (4) (taking the quantization error into account), the effective number of bits (ENOB) and the resolution for the example resistance. A slight improvement can be observed with the module CM2 in Section 3.2 regarding the simplest CM1 in Section 3.1. The module CM3 in Section 3.3 based on a front end with a latch does not perform better than the others, actually its results are worse than those achieved with CM2. However, the module CM4 that filters noisy spikes obtains better results. Finally, the capture modules that exploit both the rising and the falling edges of the clock signal to synchronize the flip-flops and carry out the average of two (CM5) of four (CM6) counts provide the best results, with resolution as low as 1.70 Ω.

**Table 1:** Precision data of the capture modules ($R_{x}$ = 7350 Ω).

	*σ*(X) (counts)	*σ*(X) (µs)	*u*(y) (µs)	ENOB (bits)	Resolution (Ω)
**CM 1**	2.14	0.043	0.043	10.98	3.54
**CM 2**	1.83	0.037	0.037	11.20	3.04
**CM 3**	2.13	0.043	0.043	10.98	3.53
**CM 4**	1.90	0.038	0.038	11.14	3.16
**CM 5**	1.31	0.026	0.027	11.67	2.19
**CM 6**	1.00	0.020	0.021	12.04	1.70

**Figure 14:**
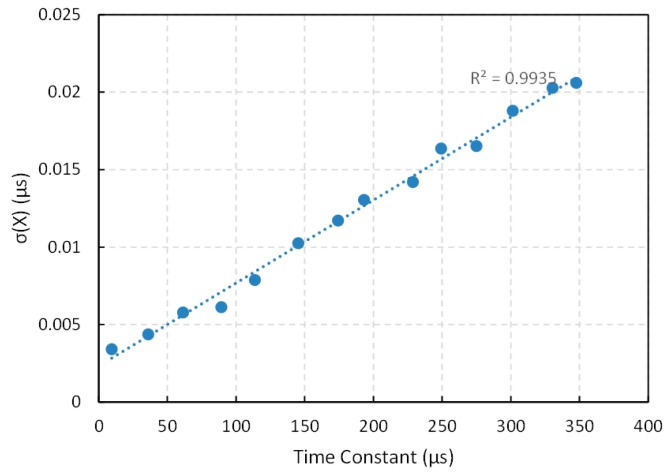
Experimental standard deviation σ(x) *versus* the time constant of the capture module in Section 3.6.

**Figure 15:**
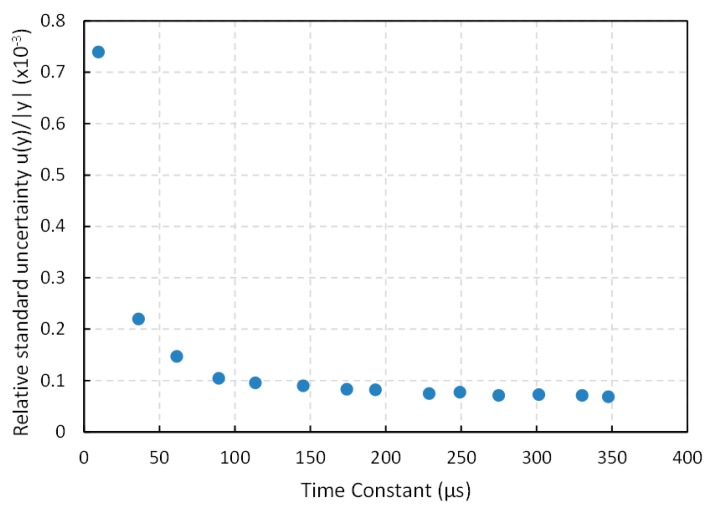
Relative standard uncertainty *versus* the time constant for the capture module in Section 3.6.

Regarding bandwidth, especially relevant if data from many sensors are acquired, it is limited by the time constant $R_{x}C$ in Equation (1) and there is a clear tradeoff with precision, because direct interfaces exploit the quantization of time so the longer the time constant the larger the precision if only the quantization error is taken into account. However, for increasing time constants, the influence of trigger noise is larger than that of the quantization error [11] and the standard deviation grows linearly with the time constant. This can be seen in Figure 14, where the standard deviation is represented *versus* the time constant for C = 47 nF and a set of resistance values (200 Ω, 762 Ω, 1300 Ω, 1890 Ω, 2400 Ω, 3070 Ω, 3680 Ω, 4100 Ω, 4840 Ω, 5300 Ω, 5820 Ω, 6400 Ω, 7000 Ω and 7350 Ω). Nevertheless, if both sources of error are added using Equation (4), the relative uncertainty behaves as shown in Figure 15. It can be observed that the relative uncertainty grows for small time constants. As mentioned above, the reason is that the quantization error is the main source or error, so if the range of time is short the precision is limited by the period of the clock signal. For increasing values of the time constant, the relative uncertainty decreases, but it changes very little for time constants above 100 µs in Figure 15. Therefore, this value can be a good compromise between precision and bandwidth. Note that the capacitor C can be chosen to set the required time constant for a given resistance range. This capacitor has to be changed if the resistance range varies to maintain the same relative uncertainty. This can require an increase in the number of bits of the counter that measure the discharge time, but does not affect the performance of the capture modules.

Relating to power consumption, its main component is that involved in the charge of the capacitor given by $\frac{1}{2}CV_{CC}^{2}f$, where f is the frequency of the charging-discharging cycle. Obviously, the larger the bandwidth the larger the power consumption, and the lower the precision because the time constant must be shorter.

With respect to the cost, Table 2 summarizes the hardware resources consumed by the capture modules. CM5 and CM6 obviously need more resources. However, this is not a serious limitation because there are a wide range of FPGA devices and more powerful ones can be chosen if the target application requires many computational resources.

**Table 2:** Hardware resources consumed by the capture modules.

	CM1	CM2	CM3	CM4	CM5	CM6
**Slices**	3	3	3	3	24	45
**4 inputs Look Up Tables**	19	19	19	19	39	99
**Flip Flops**	35	35	35	35	56	92
**Latches**	0	0	1	1	2	4

**Figure 16:**
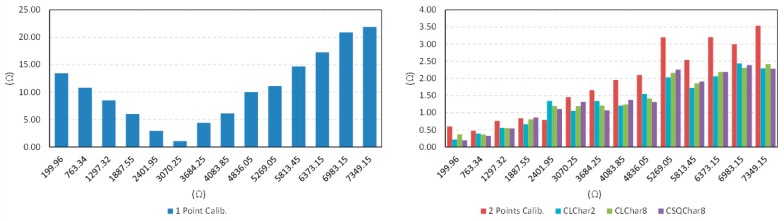
Absolute value of the error for the calibration techniques described in Section 2.1 and Section 4.

**Figure 17:**
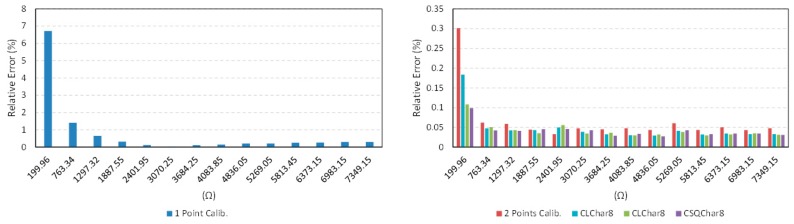
Relative error for the calibration techniques described in Section 2.1 and Section 4.

With regard to accuracy, the circuit in Figure 12 was used to perform the different calibration techniques as explained in Section 5. Table 3, Figure 16 and Figure 17 show the results. The maximum error $e_{\max}$ is that obtained for the set of resistance values in the first column of Table 3.

The best results are obtained with the proposed technique as well as with the two-point calibration. The former uses only one calibration resistor, so the cost in hardware is lower and its implementation is more compact than that of the two-point calibration. Moreover, since only one calibration resistor has to be measured per cycle of data acquisition, the bandwidth is also improved. The price to pay is the need for a previous characterization of the FPGA ports to infer the value of their input resistance, although it can be done with the same direct interface by replacing $R_{x}$ in Figure 12 with the characterization resistances and measuring the ratio $N_{x}/N_{c1}$. If a linear dependence is supposed, the slope and zero can be derived with two resistances at 15% and 85% of the range of interest (CLchar2), and also with eight different resistances in the range of interest (CLchar8). If a quadratic dependence is presumed (CSQchar8), the Equation (10) in Section 4 provides $R_{x}$ for a given $N_{x}/N_{c1}$. The results in Table 3, Figure 16 and Figure 17 do not show an improvement for the use of the techniques CLchar8 and CSQchar8 with respect to CLchar2. Therefore, since the cost of implementing CLchar2 is lower because the number of calibration resistors is lower, it is the best choice for the device of this paper.

**Table 3:** Accuracy data for the tactile sensor and different calibration techniques.

Measurand (Ω)	Maximum Absolute Error (Ω)				
	1 Point Calib.	2 Point Calib.	CLChar2	CLChar8	CSQChar8
199.96	13.44	0.60	0.22	0.37	0.20
763.34	10.80	0.47	0.39	0.36	0.32
1297.32	8.50	0.76	0.56	0.55	0.54
1887.55	6.00	0.84	0.67	0.81	0.86
2401.95	2.96	0.79	1.34	1.20	1.11
3070.25	1.08	1.46	1.05	1.19	1.32
3684.25	4.40	1.66	1.34	1.21	1.07
4083.85	6.13	1.96	1.20	1.24	1.38
4836.05	9.99	2.10	1.55	1.41	1.31
5269.05	11.10	3.20	2.03	2.16	2.26
5813.45	14.68	2.54	1.73	1.86	1.91
6373.15	17.25	3.21	2.06	2.19	2.19
6983.15	20.91	3.00	2.44	2.31	2.39
7349.15	21.90	3.54	2.29	2.42	2.28
Total Error	149.13	26.12	18.87	19.28	19.13
Max. Relative Error (%)	6.72	0.30	0.11	0.18	0.10

The resolution and accuracy tests described above were also carried out for the common PT-1000 temperature sensor. This illustrates the performance of the proposed strategies for a different resistance range, and allows it to be compared with that achieved by other reported implementations. For the range of this sensor, the resolution obtained from Equation (3) and the experimental data is 11.48 effective number of bits (0.50 Ω), which is close to that achieved for the range of the tactile sensor. Regarding accuracy, Table 4 shows the results for the one-point, two-point, and the proposed CLChar2 calibration techniques.

**Table 4:** Accuracy data for the PT-1000 temperature sensor and different calibration techniques.

Measurand (Ω)	Maximum Absolute Error (Ω)		
	1 Point Calib.	2 Point Calib.	CLChar2
759.75	4.13	0.59	0.24
876.75	2.69	1.04	0.30
948.55	1.79	1.17	0.31
1016.35	0.87	1.39	0.34
1106.65	0.87	1.78	0.34
1196.15	2.09	2.03	0.42
1296.25	3.47	2.64	0.52
1398.75	4.78	2.79	0.52
1598.25	7.42	3.50	0.62
1798.95	10.02	4.38	0.65
1891.95	11.15	4.73	0.60
2193.85	15.11	5.99	0.71
Total Error	64.40	32.03	5.57
Max. Relative Error (%)	0.69	0.27	0.04

Finally, Table 5 shows a comparison between the results obtained with the CM6 capture module and the CLChar 2 calibration technique, and those reported by other authors using direct interface of a PT-1000 sensor with other devices such as microcontrollers, CPLDs or an FPGA from a different vendor [9,12]. Note that the conditions are not the same for all the tests in Table 5, and this should be taken into account for a more thorough comparison. Firstly, the larger the voltage supply the higher the slew rate at the threshold level, which improves precision performance [9]. Secondly, the quantization noise is reduced by increasing values of the time constant [11], although the measurement time is longer and the sample rate decreases. Note that the implementation based on the microcontroller PIC16F87 conducts the two-point calibration technique, therefore it requires the measurement of three resistances per sample. Finally, the results in this paper and those in [12] are given for the worst case of the 500 samples measured, while the average of these samples is reported in [9].

**Table 5:** A comparison between this proposal and other implementations for the resistors range of PT-1000 temperature sensor ( * worst case, ** averaging 500 samples).

Device	Trigger-Calibration	Resistors Range (Ω)	Voltage Supply	RC Constant	Max. Rel. Uncertainty	Max. Abs. Error (Ω)	Max. Rel. Error (%)
PIC16F87 [12]^*^	Single Event 2 Point Cal.	825−1470	5 V	3.23 ms	0.005	0.30	0.02
PIC18F458 [9]^**^	Single Event 1 Point Cal.	817.41−2193.95	5 V	21.94 ms	0.121	8.89	0.73
CPLD EMP3064A [9]^**^	Single Event 1 Point Cal.	817.41−2193.95	3.3 V	21.94 ms	0.497	3.40	0.21
Cyclone II EP2C20 [9]^**^	Single Event 1 Point Cal.	817.41−2193.95	3.3 V	21.94 ms	0.481	26.27	2.57
Spartan 3AN^*^	CM6 CLChar2 Cal.	759.75−2193.85	3.3 V	0.37 ms	0.010	0.71	0.04

## 7. Conclusions

This paper presents circuits to implement smart direct sensor–FPGA interfaces. The proposal is especially interesting for complex systems that collect analog data from many sensors. It is suitable for resistive and capacitive sensors, though the paper shows its performance for two example resistive sensors. Since the hardware in the FPGA is configured to work in parallel, processing and data acquisition can be done in parallel to achieve high throughput and real time operation.

A number of capture modules have been proposed and implemented with the aim of improving the precision of measurement. The target is the emulation of the hysteresis present in the input buffers of other devices such as microcontrollers by adding memory and smart processing in the digital capture module. The flexibility of the storage elements in the FPGA allows us to work with flip-flops, synchronized with the rising or the falling edge of the clock signal, and to implement strategies that average the count stored by two or four capture modules working in parallel. The idea behind this approach is to filter part of the trigger noise without degrading the bandwidth. Several different design possibilities arise to use the presented capture modules, depending on the target application. If a high precision is required, for instance in the case of non-linear sensors with regions of different sensitivity, the smart module CM6 achieves a precision which is as high as 12 ENOBs for the example tactile sensor of this paper, and a measurement time of 348 µs (taking into account the charging and discharging times of the capacitor). Other capture modules can be chosen if such precision is not necessary to save resources in the FPGA.

Besides the capture modules, a calibration technique has been proposed to improve accuracy. It uses the same external resources that the one-point calibration technique based on the measurement of one known calibration resistance. However, it achieves an even better performance than that obtained by calibration with two known resistors. The bandwidth is also better because only one calibration resistance is measured per acquisition. The drawback is that a previous characterization of the input ports of the FPGA is required, since the technique actually infers the value of the input impedance of these ports. Nevertheless, there is no need for specific hardware to characterize the ports, but the same direct interface is used for this purpose. An extension of the technique makes non-linear interpolation to contemplate possible variations of the input impedance of the ports depending on the resistance to be measured, though no significant improvements have been observed for the device of this paper. The proposed technique achieves a resolution as low as 2.42 Ω for an example tactile piezoresistive sensor with a range of interest between 200 Ω and 7350 Ω. Finally, a comparison between the results obtained for a PT-1000 temperature sensor and those reported by other authors, shows improved performance.
